# Human Glanders

**Published:** 1909-03-13

**Authors:** J. M. Bernstein

**Affiliations:** Assistant Pathologist and Curator of Museum, Westminster Hospital; Lecturer in Bacteriology in the Medical School


### SPECIAL ARTICLE.

### HUMAN GLANDERS.

By J. M. BERNSTEIN, M.B., M.R.C.P., D.P.H., Assistant Pathologist and Curator of Museum, Westminster Hospital; Lecturer in Bacteriology in the Medical School.

Human glanders has appropriately been described as a loathsome and fatal disease. Often insidious in onset, it may suddenly flare up into a generalised infection as terrible in its appearances as, and more terrible in its result, than smallpox, with which, indeed, it has at times been confounded. It has been and still is so rife amongst horses—the natural hosts of B. mallei—in this country that it is difficult to explain why so few cases are met with in man.

Though comparatively rare as a human disease, it must be stated at the outset that the reported and diagnosed cases form but a small proportion of the incidence, and many cases have undoubtedly been otherwise diagnosed.

Robin*, in an exhaustive thesis, collected from the literature up to 1906 but 156 cases, and remarks on the improbability of any individual Anglo-Saxon observer encountering more than a solitary case. The Registrar-General's statistics show a mortality of one or two a year.

However, in the last few years, and coincident with the more widespread and closer association of clinical work with pathological science, many more cases have been recognised, and whilst in 1907 the Medical Officer of the London County Council reported four deaths, we know, unofficially, of at least twice this number as having occurred in London in 1908.

In the latest article on the subject†, the authors detail six cases (presenting most of the varied symptoms of the disease) which they had the good fortune personally to see and investigate from every aspect. It may be that man is but little susceptible to infection, and many instances are known of horse-slaughterers who have cut themselves during autopsies on glandrous horses without any ill effects; but, on the other hand, there are numerous instances of direct inoculation with subsequent infection amongst laboratory workers and those who have performed post-mortem examinations on human glanders. It must be noted that horses are slain before the disease becomes generalised, and before the blood is infective, whilst men die from a terminal invasion of the blood-stream by the B. mallei, and so the risks of infection are much greater.

The lesions in glanders are multifarious, and include localised intramuscular abscesses on the extremities, either single or multiple, which, after evacuation, may heal up without further symptoms; spreading subcutaneous abscesses, extending up the limbs in the course of the lymphatics, analogous to the farcy buds of the equine disease, which, after months, may cause a fatal termination by invading the blood-stream; and a chronic, slowly spreading ulcer of the skin or exposed mucosæ, which may lead to great destruction of tissue and end fatally in an acute outburst: or the disease may be acute from the onset and simulate the acute specific fevers or a pyæmic condition, and baffle diagnosis until an acute pustular exanthematous eruption appears and forecasts the fatal termination.

* Studies from Royal Victoria Hospital, Montreal, 1906.
† Observations on Human Glanders. Bernstein and Carling. *British Medical Journal*, February 6, 1909.

It is of little avail to enumerate the large number of diseases, such as smallpox, typhoid, pyæmia, syphilitic and tuberculous ulcerations, etc., etc., for which glanders has been mistaken. It is more important to remember that many different conditions give rise to abscesses, and that ulcers are caused by different agencies and pyæmia by many different organisms; it is now no longer to be expected that these conditions can be definitely diagnosed by any special characters of the pus or naked eye appearance of the ulcers. More modern methods of research have, however, facilitated diagnosis, and now, to isolate the causal agent is to clinch the diagnosis.

The disease is generally associated with severe malaise and pains in the limbs. It is advisable to bear glanders in mind when any abscess, ulcer, or severe malaise occurs in persons associated with horses, or in the families of such; for the disease has spread through several members of a family, and in one case is known to have occurred in a washerwoman who washed infected clothing.

The danger of infection is recognised to some extent amongst horsekeepers. Recently one presented himself with a pustular eruption on the wrist, and impressed with the fear that it was glanders he gave all the subjective symptoms of the disease; but a culture revealing in two or three days a typical horse-ringworm fungus, his symptoms immediately vanished.

We have already pointed out that the diagnosis, or, at least, the confirmatory diagnosis, falls within the province of the bacteriologist. The specific bacilli exist in the lesions in but very scanty numbers, and hence can be found only with difficulty, if at all, and this is further enhanced by their feeble staining affinities. But in contrast to this the cultural appearances are constant and characteristic, and within 48 hours typical luxuriant growths are obtained on suitable media. Though somewhat slower in development, the results of animal inoculation are equally constant and practically pathognomonic, and are of especial value in cases of a suspicious ulcer whose surface is so contaminated by other germs as to prevent isolation, by culture, of the specific germ: a piece of the ulcer edge inoculated subcutaneously into a guinea-pig produces characteristic enlargement of the testicles with inflammation of the tunicæ vaginales, etc., in a few days.

By far the most valuable diagnostic procedure is the mallein test. Mallein—the killed cultural product of B. mallei—has for some time been in universal use to test horses for the disease in its earliest stages. In man it is of equal importance, and, contrary to the opinion of some, it is not known to exert any ill effect. Ten to fifteen c.c. of mallein inoculated into a glandrous patient whilst the temperature is low produces in a few hours local symptoms and a marked rise of temperature through several degrees.

The duration of the disease varies within wide limits. Some chronic localised cases recover with surgical treatment, some drag on for years and then flare up into a generalised acute infection, terminating fatally, whilst some that apparently recover are said to undergo a fatal recrudescence some months or years later.

Concerning treatment there is, unfortunately, little to be said. If the localised lesion can be thoroughly excised then the prognosis is hopeful. Attempts have been made to obtain an antitoxic serum from the less susceptible animals, but up to the present without success. Vaccine therapy, which might be expected to be of some value in so chronic a condition, has been tried in several cases, but so far with indefinite results. In fact, we are compelled to admit that with our present knowledge we are in many cases only able to stand helplessly by and watch the disease drag on (more or less slowly, but nevertheless surely) to a fatal termination.

But if we can do little or nothing for the infected cases we can at least prevent further infection, and the question of prophylaxis is one that has received much attention, and offers promising results. The fact that glanders is a disease of horses which can be recognised in the early stages by the use of mallein, points to a means of eradicating it from our Island. The Board of Agriculture's Glanders and Farcy Order of 1907 has facilitated this by increasing the compensation paid to owners of slaughtered glandrous animals, so that there is little inducement to conceal the existence of the disease; further, full compensation is allowed if the diagnosis is found, post-mortem, to have been wrong. Notification is compulsory, and suspected animals are tested with mallein by the local sanitary authorities at the owner's request, and killed if they give a positive reaction.

A considerable sum has already been paid by the London County Council in compensation, and most of the large studs are being systematically tested with mallein. Hence in time it may be expected that the disease will be stamped out, and by preventing the importation of animals giving a positive mallein reaction we may hope to free ourselves of this menace to human life—a freedom that has been obtained in some countries. Meanwhile personal prophylaxis must not be ignored. Many stable hands have most uncleanly habits, and rarely trouble to wash their hands before fingering their food, and it must be recognised that many cases of infection may be due to ingestion in man as well as in horses, though some are also due to direct inoculation through neglected wounds and surface abrasions, and a few appear to have followed infection of a mucous surface by the discharges of a snorting horse.

